# Deep Convolutional Neural Network-Based Detection of Gait Abnormalities in Parkinson’s Disease Using Fewer Plantar Sensors in a Smart Insole

**DOI:** 10.3390/bios16010040

**Published:** 2026-01-04

**Authors:** Eun-Seo Park, Xianghong Liu, Han-Jeong Hwang, Chang-Hee Han

**Affiliations:** 1 Department of Computer and Information Science, Korea University, Sejong 30019, Republic of Korea; esp0117@korea.ac.kr (E.-S.P.); liuxh@korea.ac.kr (X.L.); 2 Department of Electronics and Information Engineering, Korea University, Sejong 30019, Republic of Korea; 3 Interdisciplinary Graduate Program for Artificial Intelligence Smart Convergence Technology, Korea University, Sejong 30019, Republic of Korea; 4 Digital Healthcare Center, Sejong Institute for Business and Technology, Korea University, Sejong 30019, Republic of Korea

**Keywords:** Parkinson’s disease, gait analysis, smart insole(s), convolutional neural network, plantar pressure sensor

## Abstract

Early diagnosis of Parkinson’s disease (PD) is crucial for slowing its progression. Gait analysis is increasingly used to detect early symptoms, with smart insoles emerging as a cost-effective and convenient tool for daily monitoring. However, smart insoles have practical limitations, including durability concerns, limited battery life, and difficulties in minimizing the number of sensors. In this study, we designed a novel deep convolutional neural network model for accurately detecting abnormal gaits in patients with PD using a reduced number of sensors embedded in smart insoles. The proposed convolutional neural network (CNN) model was trained on a gait dataset collected from a total of 29 participants, including 13 healthy individuals, 9 elderly individuals, and 7 patients with Parkinson’s disease (PD). Instead of combining plantar pressure data from both feet, the model processes each foot independently through sequential layers to better capture gait asymmetries. The proposed CNN model achieved a classification accuracy of 90.35% using only 8 of the 32 plantar pressure sensors in the smart insole, outperforming a conventional CNN model by approximately 10%. The experimental results demonstrate the potential of our CNN model for effectively detecting abnormal gait patterns in patients with PD while minimizing sensor requirements, enhancing the practicality and efficiency of smart insoles for real-world use.

## 1. Introduction

Parkinson’s disease (PD) is a progressive neurodegenerative disorder of the central nervous system that results from the loss of dopamine-producing neurons [1]. Primarily affecting the motor and non-motor systems, in its early stages, PD causes motor symptoms, collectively referred to as Parkinsonism [2]. Although Parkinsonism can manifest in several ways, the most iconic features of PD include tremors, muscle rigidity, bradykinesia, and postural instability [3]. Tremors and muscle rigidity are the two most common features of Parkinsonism and serve as indicators for diagnosing PD [4]. However, PD involves neurodegeneration that also affects non-motor functions. Characteristic non-motor symptoms of PD include behavioral changes and other neuropsychiatric problems expressed in the form of sleep disturbances, psychosis, loss of sense of smell, and significant mood changes [5]. The progressively deteriorating motor and non-motor symptoms of PD adversely affect patients’ quality of life (QoL) over time [6]. Patients with PD experience a honeymoon phase, an initial period of 2–5 years after PD onset, during which symptoms can be effectively controlled with medication [7]. Consequently, early diagnosis of PD is essential for effective symptom control and management during the honeymoon phase [8].

Conventional approaches to diagnosing PD depend on clinical assessments [9], neuroimaging [10], and biosignal-based analyses [11]. In general, experienced neurologists monitor the progression of motor and nonmotor symptoms over time, employ standardized rating scales, and adjust drugs over multiple consultations, yielding high diagnostic precision [12]. However, conventional diagnostic approaches require professional experts who can interpret clinical results and rely on expensive neuroimaging modalities, such as magnetic resonance imaging (MRI), positron emission tomography (PET), dopamine transporters, and single-photon emission computed tomography (DaT-SPECT) scanners [13]. Hence, an accurate diagnosis of PD occurs only in well-resourced hospitals and requires repeated long-term clinical visits [14]. When PD patients visit a hospital with symptoms, such as tremor or rigidity, a substantial loss of dopaminergic neurons has usually already occurred [15]. Initial indicators, such as anosmia, sleep disturbances, or minor alterations in gait, frequently go unnoticed, even though early recognition could enable prompt intervention in PD treatment [16]. Therefore, conventional diagnostic techniques for PD remain the clinical gold standard; however, their logistical demands and reliance on symptom-driven scheduling limit their utility in the early detection of PD [17].

Recently, several studies have reported that abnormalities in gait patterns may serve as early predictors of PD, potentially reducing the typical delays associated with clinical assessments, neuroimaging, and biosignal-based diagnoses [18,19,20]. Gait dysfunction may occur alongside or even precede the characteristic symptoms of tremors, rigidity, and bradykinesia, because dopamine degeneration affects the brain’s motor control system [21]. Among these alterations, gait asymmetry is of considerable research interest because patients with PD exhibit reduced symmetry in left–right balance, within-step timing during the stance and swing phases, and foot pressure distribution [22]. Based on this evidence, gait assessment can provide a direct and quantifiable measure of a patient’s locomotor capability, reflecting their overall range of motion. Considering spatiotemporal gait measures decline concurrently with dopaminergic degeneration [20], precise gait assessment has become a target for the early identification and longitudinal monitoring of PD symptom progression [18]. Current gait data collecting devices include video cameras, motion-tracking sensors, and specialized walkways that measure foot sole pressure [23,24]. Although these advanced systems yield highly accurate movement and force measurements, they are expensive, time-consuming to set up, require careful sensor placement, and need strict environmental controls [25]. These conditions limit their suitability for real-time or point-of-care testing, necessitate post hoc expert interpretation, and often cause patient discomfort owing to contrived test conditions [26]. Therefore, despite their high accuracy, conventional gait laboratory techniques are impractical for assessing gait abnormalities in daily life, limiting their usefulness for early diagnosis of PD.

In recent years, wearable sensor-based gait analysis systems have emerged as practical alternatives to laboratory-based video and motion capture systems, enabling continuous monitoring of PD gait signatures in daily life [27,28]. Among these wearable systems, smart insoles are particularly attractive because they fit seamlessly inside a shoe, collect plantar pressure and inertial data in real time, and operate independently of time and location constraints [29]. Smart insoles can capture subtle context-dependent gait fluctuations that often precede overt motor symptoms of PD [27,30]. Smart insoles offer significant advantages for the early detection of PD, as continuous daily-life data provide richer estimates of spatiotemporal gait variability than episodic laboratory assessments [27]. However, current smart insole systems usually have various piezoelectric pressure sensors, accelerometers, and temperature sensors, rendering the devices bulkier and placing each part under stress when walking. Consequently, durability and long-term data collection remain key challenges [31,32]. Developing machine-learning algorithms that maintain high accuracy while using fewer sensors in smart insoles is essential to improve device longevity, reduce costs, and accelerate the adoption of smart-insole technology for regular patient-focused Parkinson’s disease screening.

To overcome the limitations of smart insoles, we propose a novel deep convolutional neural network (CNN) architecture that achieves high accuracy even with a limited number of plantar pressure sensors embedded in the smart insole. Rather than treating the plantar pressure data as a single combined signal, our model independently analyzes data from each foot through sequential layers. Following the independent analysis of each foot’s data, temporal and spatiotemporal convolutional layers are applied. Using the feature maps from these layers, the proposed CNN detects unusual walking patterns observed in patients with PD considerably better than the standard model. Furthermore, the model employs a sliding-window approach, which is particularly suitable for real-time applications. The proposed method achieved an accuracy of approximately 84% (representing satisfactory performance) using far fewer pressure sensors than existing smart insole models, requiring various pressure sensors to attain comparable accuracy. Therefore, the proposed model enhances wearability and cost-efficiency of smart insoles while enabling continuous, real-world gait monitoring for the early detection and management of Parkinson’s disease.

## 2. Materials and Methods

This study follows a structured methodological pipeline consisting of five main stages. First, plantar pressure data were sourced from a publicly available smart insole dataset. Next, the raw signals underwent preprocessing and segmentation via a sliding-window approach, facilitating real-time analysis. An exploratory unit step asymmetry analysis was then conducted to characterize bilateral gait differences among healthy subjects, elderly individuals, and patients with PD. Subsequently, a spatiotemporal CNN architecture was developed to model left–right gait asymmetry based on insights from the asymmetry analysis. Finally, the model was systematically evaluated under various sensor configurations, sensor failure scenarios, and gait conditions using a stratified cross-validation framework.

Details of each stage are described in the following subsections: Section 2.1 introduces the dataset and experimental protocol, Section 2.2 discusses region-of-interest (ROI) selection, Section 2.3 elaborates on the unit step asymmetry analysis, Section 2.4 covers data preprocessing, Section 2.5 and Section 2.6 address the CNN design strategy and architecture, respectively, and Section 2.7 highlights model evaluation and training setup.

### 2.1. Dataset Information

We employed a publicly available smart insole dataset [29] to analyze the gait patterns of patients with PD. There were 29 subjects in the dataset, divided into three groups: 13 healthy adults (S), 9 elderly adults (EL), and 7 patients with PD (PD). All data were collected using a commercially available smart insole device (OpenGo Sensor Insoles, Moticon, Munich, Germany). The system had 16 plantar pressure sensors for each foot (32 for both feet), and all participants used the device for two gait tests. The first test was the walk straight and turn (WST) test, and the second was the modified timed up and go (TUG) test [29]. Both tests involved participants walking on a round trip along a 10 m straight walkway. The WST test was conducted at three different speeds (fast, normal, and slow), whereas the TUG test was conducted at a normal speed. Each test was repeated twice; however, the number of trials was adjusted for participants who had difficulty completing the tests. Gait data were collected at 100 Hz, and annotations were made to indicate two types of activities: activities of daily living (ADLs) and gait cycle events (GCEs). Because this study used a publicly available open dataset, the Institutional Review Board of Korea University determined that the study was exempt from ethical review (IRB Protocol No. 2025-0082; approval date: 9 May 2025).

### 2.2. Region of Interests (ROIs)

**Figure 1:**
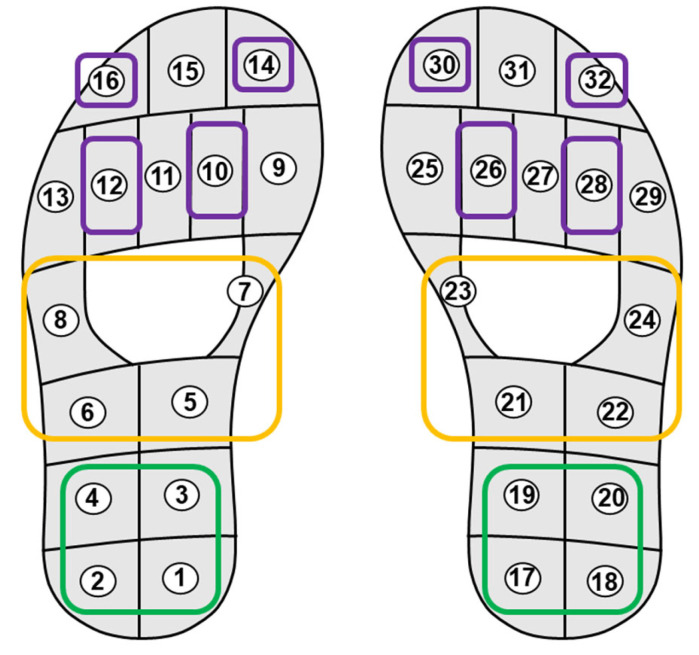
Layout of plantar pressure sensors and region-of-interest (ROI) definition. The forefoot (purple), midfoot (yellow), and hindfoot (green) regions are highlighted, with sensor IDs numbered from 1 to 16 for the left foot and 17 to 32 for the right foot.

We propose a novel CNN model to identify abnormal walking patterns in PD patients using only a limited number of plantar pressure sensors in a smart insole. We tested the effectiveness of the proposed CNN model by focusing on limited areas of the overall shape of the foot. The foot surface is divided into three anatomical regions: the hindfoot, midfoot, and forefoot, each contributing uniquely to gait cycles [33]. The hindfoot primarily provides support for landing and stability; the midfoot provides support for weight transfer and flexibility; and the forefoot plays a critical role in propulsion during the gait cycle [34]. Four pressure sensors were selected for each ROI based on sensor placements reported in previous studies [33,34]. Figure 1 shows the locations of the sensors in each ROI, where sensors for different foot regions are represented by different colors: purple for the forefoot, yellow for the midfoot, and green for the hindfoot. Each hindfoot and midfoot contained exactly four sensors; therefore, all sensors were employed. In contrast, the forefoot had more than four sensors; thus, four sensors were selected to achieve a spatial distribution similar to that of the other regions. For the forefoot region, four plantar pressure sensors were selected based on three criteria: (1) anatomical coverage of the distal forefoot during push-off and toe loading, (2) symmetric placement between the left and right feet for bilateral consistency, and (3) consistency with sensor placement strategies reported in prior plantar pressure studies [33,34]. Accordingly, sensors with IDs 10, 12, 14, and 16 were chosen for the left foot, while sensors 26, 28, 30, and 32 were selected for the right foot, as illustrated in Figure 1. This ROI setting enabled us to conduct a thorough evaluation of the areas in the foot that contributed most significantly to gait pattern analysis. Using the ROI-based method with fewer pressure sensors, we verified whether the proposed model could maintain high performance despite the reduced number of sensors.

### 2.3. Unit Step Analysis

Before developing the deep learning model, we conducted an asymmetric analysis of unit steps to investigate the gait differences among healthy subjects, elderly individuals, and patients with PD. A unit step was defined as the time from initial contact of the foot to the next contact [35]. Unit steps were extracted independently from the left and right feet based on gait phase annotations, and signals with missing or incorrect annotations were excluded. The missing values in the last extracted unit step signals were imputed using the mean of the adjacent pressure values. For bilateral comparisons, paired unit steps were identified across alternating strides, and each pair was matched without overlap to avoid duplication.

Asymmetry-related gait features were extracted using the established methods [36]. Plantar pressure signals may vary with body weight [37]; hence, features affected by body weight were excluded. The extracted features included temporal parameters (Tmax1, Tmax2, Tmin, and Tms) and variability-related parameters (NegSL, Tns, and Ts) that have been widely used in previous studies on plantar pressure and gait asymmetry [36]. For each bilateral pair, an Absolute Symmetry Index (ASI) was calculated [38], and group-level ASI values were compared using the Kruskal–Wallis test with post hoc Wilcoxon rank-sum tests and Bonferroni correction. Although this gait asymmetry analysis revealed distinct gait characteristics across the groups, it was not intended as direct input for the deep learning model. Instead, it provided physiological and biomechanical justification for the CNN design, confirming that bilateral gait asymmetry is a prominent characteristic of PD in the dataset. Consequently, a foot-wise modeling strategy was adopted in the proposed CNN architecture, allowing for independent processing of left and right foot signals to capture asymmetry-related spatiotemporal features. Unit step signals were excluded as inputs since the proposed deep learning model employs a fixed 2 s sliding window to continuously capture abnormal gait patterns during daily activities.

### 2.4. Data Preprocessing

We employed only plantar pressure sensor data acquired during the straight gait phase, excluding the turning gait phase. Turning gait phases with direction changes were excluded to minimize the possible variability due to turns. The proposed deep learning model was optimized using only slow-gait data. Previous research has shown that although gait patterns are more consistent at slow speeds, a smaller range of pressure measurements renders gait classification more difficult [39]. Accordingly, slow gait data were considered in the development of a novel deep learning model, as this condition was the most challenging scenario in the dataset.

Although the unit step method has benefits in terms of the ease of comparing similar gait patterns during offline analysis, it introduces various complicated obstacles when applied in real-time environments. For instance, real-time environments require the consistent application of sequential gait signals of a specific length, which do not necessarily correspond to unit step signals. Therefore, if the deep learning model is trained solely on unit step data, its effectiveness may be compromised in real-time applications that involve gait signals to produce usable real-time solutions. Therefore, we used a sliding-window-based method that met the requirements for real-time processing. Gait data were first divided into 2 s segments that overlapped by 50% [40]. At this point, the epochs with missing values were removed from further analyses. A total of 542 epochs were extracted from healthy participants, 525 epochs from elderly individuals, and 172 epochs from PD patients. To address the imbalance in epoch numbers across groups, we randomly selected 172 epochs from both healthy subjects and elderly individuals to match the number of epochs in the PD subject groups. This process resulted in an input vector for the deep-learning models organized in a tensor structure of 516 epochs × 32 sensors × 200 time samples, which pooled data from healthy individuals, elderly individuals, and PD patients. Furthermore, we trained and tested the performance of the models under diverse gait paradigms (fast, normal, and TUG), in addition to the slow gait paradigm, using input vectors with the same structure. Although similar preprocessing procedures were performed for each gait paradigm, they had different epoch sizes owing to differing measuring times as well as distinct inherent characteristics of distinct gait types (258 epochs for fast, 630 epochs for normal, 516 epochs for slow, and 762 epochs for TUG).

### 2.5. Model Design Strategy

To accurately detect abnormal gait patterns in patients with PD, we designed a deep CNN model with specialized convolutional layers. The accuracy degradation typically associated with a reduced number of plantar pressure sensors in a smart insole was minimized by structuring these convolutional layers to effectively capture the temporal or spatial features of gait signals while accounting for the distinct gait patterns of each foot.

**Figure 2:**
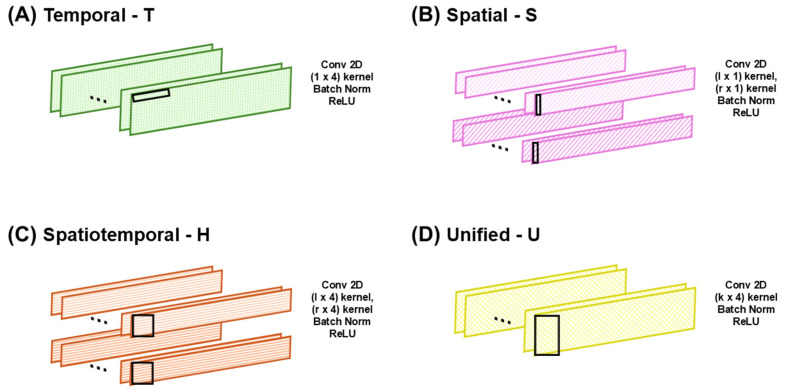
Types of convolutional layers in the proposed convolutional neural network (CNN) architecture: (**A**) temporal, (**B**) spatial, (**C**) spatiotemporal, and (**D**) unified. Symbols *l* and *r* denote left and right foot inputs, respectively. The black rectangles indicate the kernel size of the CNN.

The proposed CNN model employs four specific convolutional layers (Figure 2): (a) a temporal (T) layer, specifically developed to capture temporal dynamics; (b) a spatial (S) layer, which independently processes each foot to capture spatial features; (c) a hybrid (H) layer, designed to represent both temporal and spatial features simultaneously; and (d) a unified (U) layer, which jointly analyzes gait patterns from both feet. All possible combinations of these convolutional layers according to the number of layers in the CNN model were systematically evaluated to define the optimal CNN architecture with the best classification performance using the predesigned convolutional layers, T, S, H, and U. The optimal model configuration was determined by progressively increasing the number of layers until saturation was achieved. Considering that gait involves alternating movements of both feet, which vary based on individual health and motor function [41,42], the CNN layers were designed to prevent overlap between the left- and right-foot data by applying each foot’s data to two independent layers, thereby enabling independent analysis of each foot. This design strategy enables the effective learning of spatial features based on sensor placement within the smart insole. In the first application, the S and H layers process the left- and right-foot data separately through independent layers, after which the outputs are concatenated along the temporal dimension. As the data are processed independently before concatenation, the model can flexibly accommodate cases where the number of sensors differs between the feet. The S and H layers may be applied up to two times; in their second application, they jointly process gait patterns from both feet without input separation, thereby capturing the integrated bilateral features. The U layer, adapted from the model proposed by Lee et al. [43], simultaneously processed sensor data from both feet and captured the combined spatiotemporal features. However, it can only be employed once because further applications would alter the data dimensions and disrupt subsequent processing. Each CNN layer employs purposefully selected kernels and strides to reflect its functional roles. A 2D convolutional structure was employed, enabling the simultaneous consideration of both the spatial and temporal dimensions. The spatial kernel dimensions were set to either k/2 or k, where k represents the number of sensors, and the temporal kernel size was determined as 4. The stride length was carefully chosen to avoid overlap between the feet, and a temporal stride value of 2 was selected. The kernel and stride parameters were selected empirically by testing multiple candidates, and the reported configuration yielded the highest classification accuracy.

After determining the proposed CNN architecture, we designed a control model for comparative analysis. The control model employed an identical number of layers to those of the proposed CNN model, but simultaneously analyzed both feet by implementing a UTTTTT layer sequence. This control model was designed based on a conventional model reported in a previous study [43] that demonstrated excellent performance across different gait patterns using a CNN architecture. Notably, the control model employed a different number of layers compared with the original model to ensure a fair evaluation using the proposed CNN architecture. By comparing the performance of the two models, we evaluated the effectiveness of independently analyzing each foot to improve the model performance.

### 2.6. Proposed CNN Model Architecture

**Figure 3:**
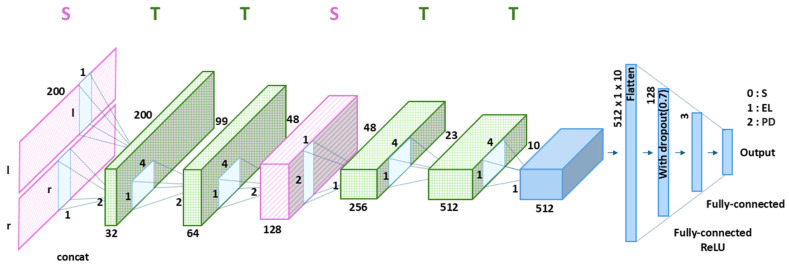
Architecture of the proposed CNN model with the optimized STTSTT layer configuration, where S and T denote spatial and temporal convolutional layers, shown in pink and green, respectively. Symbols *l* and *r* denote the left and right foot inputs, respectively. Black S, EL, and PD represent a healthy subject, an elderly individual, and a patient with Parkinson’s disease, respectively.

The proposed CNN model employs six 2D convolutional layers arranged in the STTSTT order (Figure 3). In the figure, *l* and *r* represent the number of sensors on the left and right feet, respectively. The full-sensor version used 32 sensors on both feet, whereas the ROI-based version employed only 8 sensors on both feet. The S layer, which is the first convolutional layer, examines the data from each foot separately to identify unique asymmetric spatial gait features. The second and third convolutional layers, which are both T layers, capture the gait patterns based on time-based gait features. The fourth convolutional layer, an S layer, combines the spatial features of both feet to analyze their combined gait. The next two convolutional layers, which are both T layers, further enhance the extracted gait features by focusing on temporal dynamics. Each convolutional layer underwent batch normalization and ReLU activation. This enhances model stability during training and introduces nonlinearity to enhance feature representation. After passing through all convolutional layers, the extracted feature maps were flattened into a 1-dimensional feature vector (512 × 1 × 10). This flattened vector is passed through two fully connected layers. The first fully connected layer consisted of 128 output nodes with a ReLU activation function, followed by a dropout layer with a rate of 0.7 to prevent overfitting and vanishing gradient. The second fully connected layer consisted of 3 output nodes corresponding to the three classification categories: healthy subjects, elderly individuals, and patients with PD. Finally, a softmax activation function was applied to the output layer to produce the classification probabilities. The proposed CNN model was designed to accurately capture the spaciotemporal features related to the gait cycle by analyzing each foot separately and simultaneously, considering both temporal and spatial features. Therefore, the model remains effective even with a limited number of sensors.

### 2.7. Model Evaluation and Training Setup

A stratified 10-fold cross-validation was employed to evaluate the generalized classification performance of the proposed CNN and control models. In each fold, the dataset was divided into training (72%), validation (18%), and testing (10%) subsets. The validation subset was used for hyperparameter tuning and early stopping, while the testing subset was used exclusively for performance evaluation. All reported results correspond to the average metrics across the 10 folds.

Cross-validation was conducted at the window level using sliding windows extracted from continuous gait recordings rather than on a subject-wise basis. To mitigate potential information leakage from overlapping windows, window generation preserved the temporal order of the signals, ensuring no overlap between the training, validation, and testing subsets within each fold.

The cross-validation was conducted at the window level rather than on a subject-wise basis, using sliding windows extracted from continuous gait recordings. To mitigate potential information leakage arising from overlapping windows, window generation preserved the temporal order of the signals, and no overlapping windows were shared between the training, validation, and test subsets within each fold.

First, we evaluated the classification performance of the proposed CNN model using all 32 sensors to verify whether the proposed CNN architecture could achieve a performance comparable to that of existing deep learning models. F1-score, sensitivity, specificity, and accuracy were calculated as performance measures. In addition, confusion matrices were obtained to monitor the class-specific classification performance for each group (healthy individuals, elderly individuals, and patients with PD). After validating the classification performance of the proposed CNN model with all sensors, we confirmed the model performance using a limited number of sensors within each ROI, specifically 8 sensors per ROI. This evaluation aimed to verify whether the proposed CNN model can maintain reasonable accuracy despite using significantly fewer sensors.

During the training process, we employed Kaiming initialization for an efficient weight initialization [44]. An Adam optimizer with a learning rate of 0.0005 was employed to optimize the training process. Cross-entropy loss was selected as the loss function, which is suitable for multiclass classification problems. A batch size of 32 was employed for all the experiments. Training was conducted for a minimum of 20 epochs and a maximum of 50 epochs, with an early stopping criterion activated when the validation loss increased after 20 epochs. The maximum number of epochs was set as 50. All hyperparameters, including learning rate, batch size, and number of epochs, were tuned in the preliminary experiments. The selected hyperparameters were largely consistent with the values commonly reported in previous studies, and preliminary experiments confirmed that varying these parameters within a reasonable range did not yield substantial differences in performance.

Finally, to further assess the robustness of the proposed CNN model, we simulated sensor failures by systematically removing sensors 1–30 from the full 32-sensor insole configuration. For single sensor failures, all 32 possible scenarios were tested. For two or more sensor failures, 100 random sensor combinations were sampled, and the average classification accuracy across these models was reported. For each failure scenario, the model was retrained and evaluated to examine the performance degradation as the number of failed sensors increased. For reproducibility, random seeds were fixed across the PyTorch, NumPy, and CUDA operations. The model was implemented using Python version 3.11.7, PyTorch version 2.2.1+cu121, CUDA version 12.1, and NumPy version 1.26.4.

## 3. Results

### 3.1. CNN Model Optimization

Table 1 presents the experimental results from the optimization process used to develop the proposed CNN model with optimal classification performance. The results were obtained by training the model using slow-gait data only. In particular, the table shows the optimal combination of convolutional layers that achieved the highest average accuracy as the number of convolutional layers was increased from one to seven. Among the deep-learning models with different layer sizes, the STTSTT model with six layers achieved the highest average accuracy of 92.28%. The average accuracy (Avg ALL) gradually increased with the number of layers, but declined when the network reached seven layers. Based on these results, the six-layer STTSTT model was selected as the final model with the best performance. Overall, the models that began with either an S (spatial) or H (hybrid) layer demonstrated higher accuracy. Both the S and H layers independently process gait signals from the left and right feet, enabling the model to separate and learn foot-specific characteristics in the initial stage, thereby facilitating more effective spatiotemporal feature extraction in subsequent layers. In terms of the ROI, the accuracy of the hindfoot was higher than that of the other two regions (94.96% for the hindfoot, 88.54% for the midfoot, and 87.55% for the forefoot); however, as the number of layers increased, the classification accuracy of the forefoot rapidly improved. Previous studies reported that plantar pressure signals in the hindfoot tend to be more loaded and repeatable than those in the midfoot or toes, enhancing discriminability [45,46,47]. Our results are consistent with these observations.

### 3.2. Results of Unit Step Analysis

**Figure 4:**
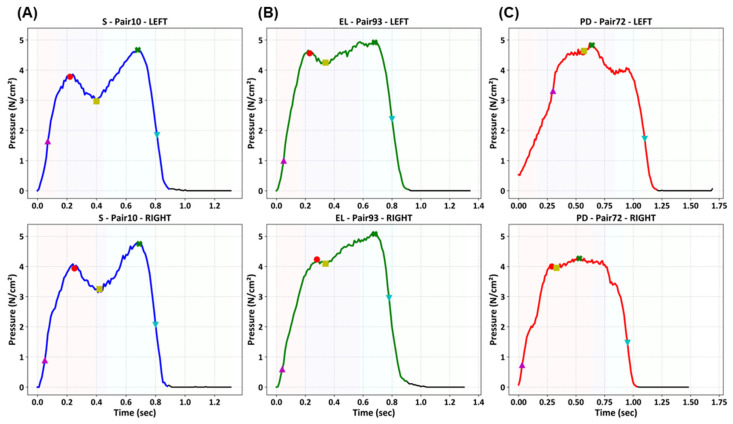
Representative paired unit step plantar pressure signals from a healthy subject (S), an elderly individual (EL), and a patient with PD. The top and bottom rows correspond to the left and right foot signals, respectively, while the columns represent the different subject groups. Markers indicate temporal and variability-related gait features for asymmetry analysis.

Before performing deep learning analysis, we conducted an asymmetric gait analysis of unit steps to confirm the differences in gait patterns among healthy subjects, elderly individuals, and patients with PD. Figure 4 shows representative examples of paired unit steps from the left and right feet of each group (healthy subject = S; elderly individuals = EL; and patients with PD = PD). In healthy individuals, the paired pressure curves showed a generalized and well-aligned shape, which is characteristic of a normal gait cycle. A typical plantar pressure curve during the stance phase follows an M-shaped trajectory [48], with two peaks separated by a minimum corresponding to load acceptance and push-off, which was observed in healthy adults. Elderly individuals exhibit noticeable irregularities with left and right foot curves differing in magnitude and timing, reflecting an age-related decline in muscle strength and motor coordination [49]. These distortions are more pronounced in patients with PD. The paired curves deviated substantially from the typical gait shape, indicating greater asymmetry and inconsistent step durations across feet.

Table 2 presents the results of the statistical analyses of the bilateral asymmetry features across multiple gait metrics. Significant group differences were observed in Tmax1, Tmax2, Tms, NegSL, Tns, and Ts. Among these, Tmax2, Tms, NegSL, and Tns exhibited asymmetry values that significantly differed between patients with PD, healthy subjects, and elderly individuals. These results confirmed that the dataset captured clear intergroup differences in bilateral gait asymmetry, with PD patients indicating the most pronounced deviations from normal gait symmetry.

The markers in Figure 4 indicate the extraction points of the gait analysis metrics, including temporal parameters (e.g., Tmax1, Tmax2, and Tms) and variability-related parameters (e.g., NegSL, Tns, and Ts), defined according to established methods [36]. The asymmetric patterns observed in PD patients are consistent with their clinical manifestations, such as impaired coordination and asymmetric motor control, and are consistent with previous studies that have reported similar gait asymmetry in PD [22].

### 3.3. Classification Results Using All Sensors

**Figure 5:**
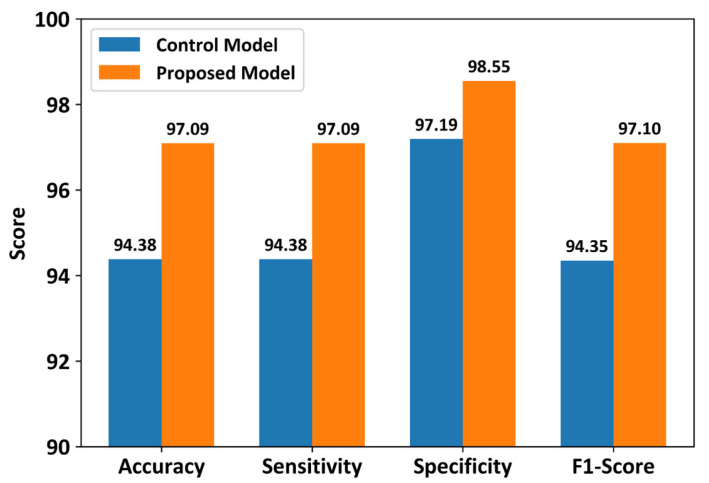
Performance comparison between the proposed CNN and control models using all 32 plantar pressure sensors. Results represent average test performance across stratified 10-fold cross-validation using slow-gait data.

Figure 5 shows the classification performance of the control and proposed models using all 32 sensors in the smart insole. The control model achieved a classification accuracy of 94.38%, whereas the proposed model achieved a classification accuracy of 97.09%. The proposed model showed approximately 3% higher classification accuracy than the control model. Additionally, other evaluation metrics, such as sensitivity, specificity, and F1-score, of the proposed CNN model were higher than those of the control model. These results confirm that the proposed model outperformed the control model when the full-sensor configuration of the smart insole was used. However, statistical analysis using the Wilcoxon signed-rank test indicated that the differences between the control and proposed models were not statistically significant for any of the evaluated metrics.

**Figure 6:**
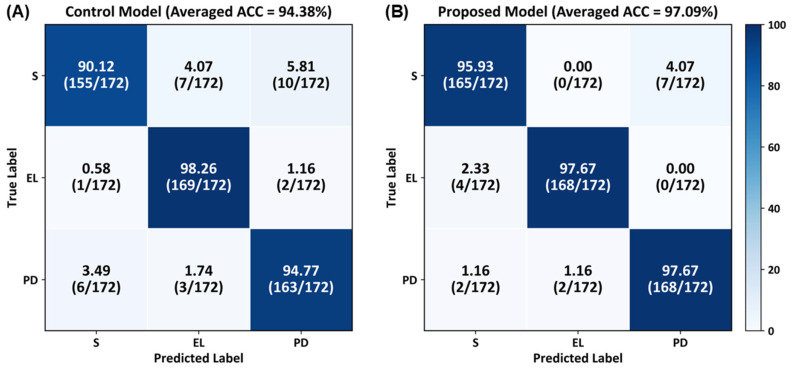
Confusion matrices illustrating class-specific classification performance for healthy subjects (S), elderly individuals (EL), and patients with PD, averaged over stratified 10-fold cross-validation using slow-gait data.

Figure 6 presents the confusion matrix results for class-specific performance across healthy individuals, elderly individuals, and patients with PD. In the control model, the classification accuracies for the healthy, elderly, and PD groups were 90.12%, 98.26%, and 94.77%, respectively. In the proposed CNN model, the classification accuracies for the healthy, elderly, and patients with PD groups were 95.93%, 97.67%, and 97.67%, respectively. The proposed model achieved higher accuracies for the healthy and PD groups compared with the control model; both models achieved similar classification accuracies for the elderly group.

### 3.4. Classification Results by ROIs with Fewer Sensors

**Figure 7:**
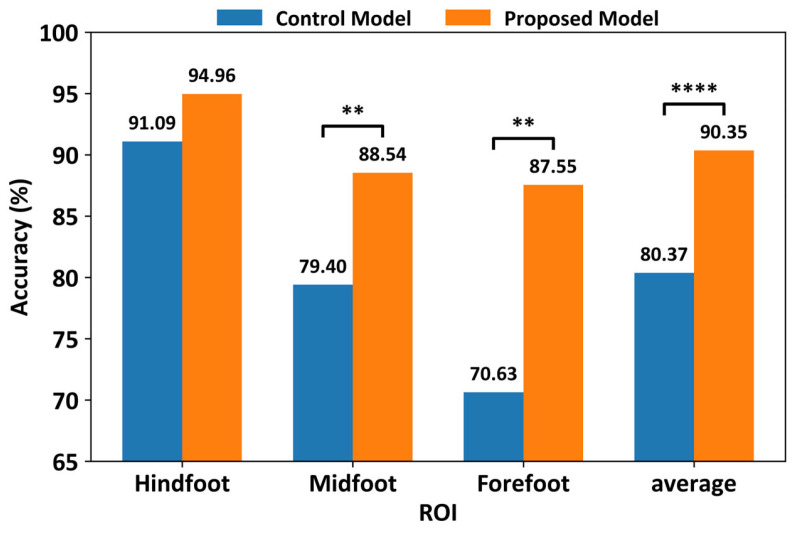
Comparison of classification performance between the proposed CNN and control models under ROI-based reduced-sensor configurations (8 sensors per ROI). Results represent average test accuracy across stratified 10-fold cross-validation using slow-gait data (** *p*-value ≤ 0.01; **** *p*-value ≤ 0.0001).

Figure 7 shows a comparison of the classification performances using only a limited number of sensors in each ROI. The control model exhibited classification accuracies of 91.09% for the hindfoot, 79.40% for the midfoot, and 70.63% for the forefoot, whereas the proposed CNN model achieved classification accuracies of 94.96% for the hindfoot, 88.54% for the midfoot, and 87.55% for the forefoot. When the number of sensors in the smart insole was decreased, the control and proposed models showed average classification accuracies of 80.37% and 90.35%, respectively. Although the classification accuracy of ROIs with fewer sensors (8/32 sensors = 25%) was slightly lower than that of the model using all sensors, the proposed model showed an approximately 10% higher classification accuracy across all ROIs (3.87% for the hindfoot, 9.14% for the midfoot, and 16.92% for the forefoot) compared to those of the control model. Statistical analysis using the Wilcoxon signed-rank test revealed that the performance improvements of the proposed model over the control model were statistically significant for the forefoot and midfoot regions, as well as for the averaged accuracy across ROIs. For the hindfoot region, although the proposed model consistently achieved higher accuracy, the difference did not reach statistical significance. These results indicate that the proposed model provides robust performance achieved under reduced-sensor configurations.

### 3.5. Classification Results Under Multiple-Sensor Failures

**Figure 8:**
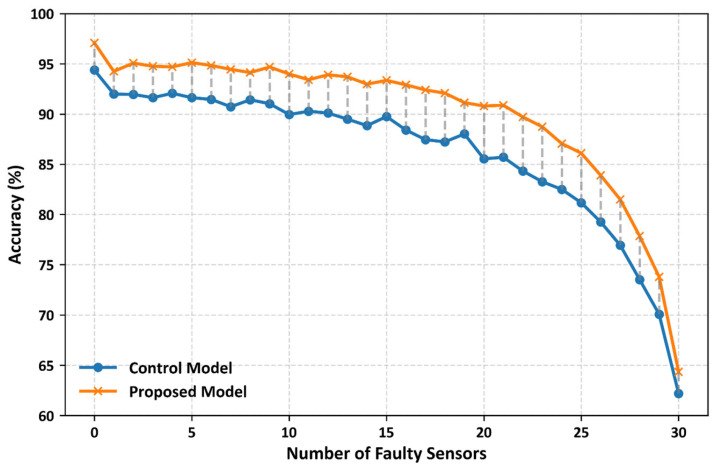
Classification accuracy of the proposed and control models under simulated multi-sensor failure scenarios. Results represent average test performance across stratified 10-fold cross-validation, demonstrating robustness to increasing sensor failures.

Figure 8 illustrates the classification performance of the control and proposed CNN models under the simulated multiple-sensor failure scenarios. In this experiment, we systematically removed sensors 1–30 from the full set of 32 pressure sensors in a smart insole. The results showed that the proposed CNN model consistently outperformed the control model under all the failure conditions. On average, the proposed CNN model achieved approximately 3% higher classification accuracy than the control model. The largest difference was observed when 23 sensors failed, and the accuracy of the proposed model was 3.84% higher. These findings highlight the robustness of the proposed model, demonstrating that it consistently delivers superior classification performance even as the number of failed sensors increases.

### 3.6. Classification Results by Different Gait Types

To evaluate the performance of abnormal gait detection in PD patients walking with different gait styles, we assessed the classification performance of the proposed model using data collected from various gait experimental paradigms, including fast, normal, slow, and TUG walking. Table 3 presents the classification performance obtained when the proposed model was evaluated using data from different gait paradigms. The average classification accuracies for each ROI were 89.18% for fast walking, 88.97% for normal walking, 92.04% for slow walking, and 82.56% for fast, normal, slow, and TUG walking, respectively.

As aforementioned, the deep learning model in this study was developed using only slow walking data because slow walking data represent the most challenging scenario where abnormal gait patterns are relatively less pronounced compared to other gait paradigms [39]. As shown in Table 3, despite being developed using only slow-walking data, the proposed model achieved promising classification accuracies of above 80% in the fast, normal, and TUG walking paradigms. Although there was a slight decline in performance, the model demonstrated the ability to maintain reasonable accuracy even under gait conditions that were not employed for training. These findings indicate that the proposed model possesses a certain degree of generalizability, enabling it to detect abnormal gait patterns in slow-walking scenarios as well as across different gait paradigms.

## 4. Discussion

In this study, we investigated the feasibility of detecting PD–related gait abnormalities using a smart insole-based deep CNN with fewer pressure sensors. The proposed architecture was designed to capture spatiotemporal gait characteristics, emphasizing left–right gait asymmetry, a key indicator of PD. We assessed the effectiveness of our model by comparing its classification performance with that of a conventional CNN architecture that does not explicitly model gait asymmetry [43]. The following sections discuss the motivation for our approach, architectural design, performance characteristics, robustness, and identified limitations.

### 4.1. Motivation and Practical Challenges in Wearable Gait Analysis

Gait analysis using wearable devices has the advantage of collecting data in real-time and analyzing gait patterns in natural environments [24,50]. In this context, real-time analysis refers to the continuous and timely processing of gait signals during everyday activities, moving beyond just automated diagnosis. Such real-time monitoring enables the longitudinal assessment of gait patterns, supporting clinical screening, rehabilitation tracking, and treatment evaluation in natural settings. This perspective is consistent with prior research on wearable gait analysis. For example, Anderson et al. proposed a novel wearable framework that combined IMU and smart insole data to continuously monitor gait and balance under both clinical and free-living conditions [50]. Recently, Nassajpour et al. compared IMU-based wearables, depth cameras, and pressure walkways and demonstrated that foot-mounted IMUs can provide accurate gait measurements in natural environments while maintaining portability and real-time usability [24]. However, wearable devices, such as smart insoles, tend to employ numerous sensors to improve accuracy, which can cause issues, such as reduced durability, shorter battery life, and discomfort during long-term use [31,32,51]. In particular, the greater the number of sensors, the more burdensome the device becomes for prolonged wear, and managing the power consumption becomes a challenge, hindering long-term usage. Such issues have motivated research efforts to improve battery longevity using both hardware and software approaches. Depari et al. proposed a self-powered piezoresistive smart insole equipped with a low-power BLE module, where optimized sensor matrices and energy-harvesting mechanisms extended the autonomy of the device to approximately nine days without compromising gait signal fidelity [52]. Using a software-oriented approach, Chen et al. introduced AdaSense, an adaptive sensing framework that dynamically adjusts the sampling rate of sensors based on user activity [53]. By lowering the sampling frequency when high-resolution data were unnecessary, their method reduced energy consumption by nearly 69% while maintaining high classification accuracy. These studies highlighted two complementary strategies: hardware-level energy harvesting and software-level sampling optimization, both of which aim to extend the operational lifespan of wearable gait analysis devices. In this study, we propose a software-based solution to overcome the aforementioned limitations of smart insole devices. Contrary to previous studies [52,53], we introduced a novel deep-learning model that can achieve reliable classification performance with fewer sensors. To accomplish this, the foot was divided into three anatomical regions: hindfoot, midfoot, and forefoot, with each region utilizing only 25% of the total number of sensors. This minimal sensor configuration enabled the development of a deep CNN model that maintained excellent classification accuracy while offering practical advantages, such as simplified hardware, reduced manufacturing costs, and improved comfort for prolonged use.

### 4.2. CNN Architecture Design and Comparative Analysis

For the past decade, various deep learning models have been developed to improve the classification performance of smart insoles [43,54,55]. Lee et al. transformed plantar pressure sensor data into unit step inputs and applied a deep CNN that fused the temporal and spatial axes into a 2D representation while processing all sensors simultaneously [43]. Although effective at distinguishing gait types, this approach relies heavily on unit step segmentation and whole-foot integration, thereby limiting its adaptability to real-time monitoring. Mun and Choi proposed an LSTM-based model that reconstructed full plantar pressure maps from a sparse input of eight sensors per foot, demonstrating that recurrent networks can infer high-resolution distributions from reduced inputs. However, the focus was on reconstruction rather than disease-specific gait modeling [55]. Park et al. introduced a temporal convolutional neural network (TCNN) to detect freezing-of-gait events in PD using insole pressure signals, employing stacked causal (dilated) convolutions to capture multi-scale temporal dependencies; although effective for freezing of gait detection, it targeted a specific symptom rather than general gait asymmetry [54]. Therefore, although previous deep-learning models have improved the classification performance of abnormal gait patterns, most conventional studies have concentrated on developing deep learning models without incorporating features specialized for gait asymmetry in patients with PD. Moreover, most prior approaches relied on complex input transformations (e.g., unit step segmentation) or dense/full-sensor configurations, rendering them less suitable for lightweight real-time applications. The proposed model was optimized using a six-layer STTSTT architecture and achieved the highest average accuracy (92.28%) in the layer combination comparison (Table 1). As shown in Table 1, the models beginning with an S or H layer consistently demonstrated superior performance. This can be interpreted as due to the independent processing of the left- and right-foot signals in the early stages, which directly reflects gait asymmetry, a representative hallmark of PD. In the STTSTT configuration, the first S (spatial) layer independently extracts foot-specific spatial features, thereby capturing asymmetry, whereas subsequent T (temporal) layers progressively learn the temporal characteristics of the gait. The second S layer integrates bilateral spatial information, and the final T layers incorporate longer-range temporal dependencies. This two-step approach, which first separates and learns asymmetry and then integrates bilateral features, enabled the STTSTT model to extract detailed and structured gait representations, thereby demonstrating its superior performance compared with other layer combinations. Furthermore, 10-fold cross-validation confirmed the strong generalization capability of the proposed model, suggesting that it can robustly detect abnormal gait patterns under diverse gait conditions.

### 4.3. Classification Performance and Robustness Analysis

As shown in Figure 5 and Figure 7, the proposed STTSTT CNN achieved 97.09% accuracy with all sensors and an average accuracy of 90.68% when employing only eight sensors per ROI (four per foot). In particular, the hindfoot ROI recorded the highest accuracy (94.95%), indicating that this region provides discriminative information for detecting abnormal gait in patients with PD. This is biomechanically plausible because the hindfoot governs initial ground contact and early load acceptance, which are critical for stability during gait. Several prior studies support this finding: Santos et al. noted in a systematic review that the hindfoot consistently indicates higher reliability in plantar-pressure measurements compared to other regions [32]; Kimmeskamp and Hennig reported distinctive heel-to-toe alterations in PD patients that emphasized the hindfoot’s role in initial contact and stability [45]; Leyh and Feipel found significant sex- and velocity-related differences in hindfoot peak pressure and contact area, suggesting its reliability for gait assessment [56]; and a clinical study reported that heel-strike pressure–time integral and contact area metrics were significantly altered in PD patients, reinforcing the diagnostic value of hindfoot signals [57]. Consistent with these findings, we observed that the hindfoot outperformed the midfoot and forefoot, strengthening the prioritization of rearfoot information in PD gait analytics. To the best of our knowledge, ROI-based PD classification with a minimal sensor configuration remains underexplored; thus, our results suggest that concentrating sensor placement in the hindfoot can preserve diagnostic accuracy while reducing hardware burden, supporting the development of miniaturized, low-power smart-insole systems for PD screening and monitoring.

In addition, the sensor failure simulation results (Figure 8) demonstrate that the proposed model consistently outperforms the control model under all conditions. On average, it maintained a performance advantage of approximately 3%; even in the case of up to 23 failed sensors, the proposed model achieved 3.84% higher accuracy. Under a single-sensor failure, the proposed model achieved 95.56% accuracy, clearly surpassing that of the control model (92.05%). These findings suggest that the proposed model maintains strong robustness against hardware instabilities, such as sensor malfunctions or data loss. In particular, wearable devices, including smart insoles, are prone to sensor degradation, poor contact, and battery depletion during long-term use [31,32,33,51]. The results of this study indicate that stable abnormal gait detection can be achieved even under realistic constraints, enhancing the feasibility of deploying the proposed approach in both clinical and daily life environments.

Furthermore, the proposed model maintained classification accuracies above 80% across the fast, normal, and TUG gait paradigms despite being trained solely on slow-gait data (Table 3). Although some performance degradation was observed, the model still possessed a certain level of generalizability, enabling it to detect abnormal gait patterns even under conditions excluded in the training. The results can be interpreted from several perspectives. First, individuals tend to exhibit more stable gait patterns at walking speeds that are familiar and comfortable to them [58,59]. Consequently, under normal gait and TUG conditions, discriminative features for identifying abnormal gait may be relatively reduced, yielding a greater decline in accuracy. Second, the slow-gait data used in this study had narrower pressure ranges and less distinct gait patterns, rendering it the most challenging condition for classification [39]. As the model was optimized under such demanding conditions, it probably secured robustness, enabling it to maintain a stable performance across diverse gait paradigms. In addition, when combined with the sliding-window method applied during preprocessing, the proposed approach demonstrates its suitability for controlled laboratory experiments, as well as for real-time monitoring of abnormal gait in daily life environments.

The confusion matrix results presented in Figure 6 demonstrate that the proposed model achieved an overall better performance than the control model in classifying gait patterns among healthy individuals, elderly participants, and patients with PD. Notably, the proposed model showed significant improvements in the healthy and PD groups, indicating its ability to capture subtle differences between normal and abnormal gaits. However, the elderly group exhibited the highest classification accuracy, which may be related to the dataset characteristics. While the healthy group included female participants, both the elderly and PD groups consisted exclusively of male participants. As previous studies have reported sex-related differences in gait parameters, such as stride length, walking speed, and plantar pressure distribution [48,56], the inclusion of females in the healthy group may have increased intra-group variability, rendering classification relatively difficult. Moreover, variability in disease progression and gait impairment severity among patients with PD could have reduced the classification consistency in that group. In contrast, the elderly group, composed solely of males, probably displayed more homogeneous gait characteristics, which resulted in the highest classification accuracy.

### 4.4. Limitations and Challenges

Despite our promising results, several limitations exist. First, the publicly available dataset used in this study included a relatively small number of participants, particularly patients with PD, which may limit the generalizability of our findings. Second, all data were collected using a single commercial smart insole device (Moticon SCIENCE), limiting validation across different sensor hardware or insole designs. Future work should involve larger and more diverse cohorts as well as data collected under various gait conditions in clinical settings to enhance robustness and generalizability. Additionally, our evaluation employed window-level cross-validation rather than subject-wise validation, which may have led to optimistic performance estimates. Demographic factors, such as sex imbalance across groups and differences in age and BMI distributions, were not controlled for in this study, which may have influenced gait characteristics and classification performance. Finally, although we designed the proposed framework for real-time use, challenges related to battery life and processing speed were not empirically evaluated. Addressing these issues by developing lightweight model architectures and improving energy efficiency will be important directions for future work.

## 5. Conclusions

In this study, we proposed an asymmetry-aware deep CNN framework for detecting PD-related gait abnormalities using plantar pressure data from smart insoles. By explicitly modeling left–right foot asymmetry and employing a sensor reduction strategy based on ROI, the proposed model achieved robust classification performance with fewer sensors. Experimental results show consistent improvements over a conventional CNN model across full-sensor, reduced-sensor, and sensor failure scenarios, as well as across multiple gait conditions. These findings suggest that asymmetry-focused modeling can enhance the robustness and practicality of smart insole-based gait analysis systems. The proposed approach may facilitate long-term gait monitoring and provide a complementary clinical assessment tool for PD in future real-world applications.

## Figures and Tables

**Table 1:** Average test accuracy from stratified 10-fold cross-validation using only slow-gait data, focusing on systematic optimization of model architecture via progressive evaluation of convolutional layer configurations.

Layer No.	Layer Type	Accuracy (%)					
		All	Hindfoot	Midfoot	Forefoot	Average	Average (ROI Only)
1	U	95.13	86.27	76.36	59.96	79.43	74.19
2	HS	90.85	86.99	80.78	78.80	84.36	82.19
3	STS	89.96	91.08	87.18	74.95	85.79	84.40
4	SHTT	93.37	92.06	86.99	85.01	89.36	88.02
5	HSTTT	92.10	92.24	88.19	86.77	89.82	89.06
6	STTSTT	97.09	94.96	88.54	87.55	92.04	90.35
7	STTTHTT	95.51	91.27	88.55	86.55	90.47	88.79

T = temporal; S = spatial; H = spatiotemporal; U = unified.

**Table 2:** Statistical analysis of bilateral gait asymmetry features derived from unit step analysis for healthy subjects (S), elderly individuals (EL), and patients with Parkinson’s disease (PD).

Feature Types	Kruskal-Wallis	Wilcoxon Rank-Sum with Holm Correction		
	*p*-Value	S vs. EL	S vs. PD	EL vs. PD
Tmax1	<0.001	<0.001 ***	0.460	0.104
Tmax2	<0.001	0.001 **	<0.001 ***	0.035 *
Tmin	0.026	0.068	0.563	0.068
MaxSL	0.295	—	—	—
Tms	0.019	0.532	0.030 *	0.030 *
NegSL	0.007	0.146	0.044 *	0.008 **
Tns	<0.001	<0.001 ***	<0.001 ***	0.034 *
Ts	0.002	0.006 **	0.009 **	0.760

*** *p*-value ≤ 0.001; ** *p*-value ≤ 0.01; * *p*-value ≤ 0.05.

**Table 3:** Average test accuracy of the proposed CNN model across different gait paradigms. For each gait paradigm, a separate stratified 10-fold cross-validation procedure was conducted using data from that specific gait condition, where the model was trained and tested within the corresponding dataset. The reported values represent the average test accuracy across the 10 folds.

Gait Type	Accuracy (%)				
	All	Hindfoot	Midfoot	Forefoot	Average
Fast	91.03	90.20	93.37	82.12	89.18
Normal	93.65	91.11	87.78	83.33	88.97
Slow	97.09	94.96	88.54	87.55	92.04
TUG	89.83	84.44	83.42	72.52	82.56

## Data Availability

The data presented in this study are publicly available at *The Smart Insole Dataset* repository: https://bmi.hmu.gr/the-smart-insole-dataset (accessed on 1 December 2025) in accordance with the dataset’s access conditions.

## Abbreviations

The following abbreviations are used in this manuscript:

PD	Parkinson’s disease
QoL	Quality of life
MRI	Magnetic resonance imaging
PET	Positron emission tomography
DaT-SPECT	Dopamine transporters and single-photon emission computed tomography
CNN	Convolutional neural network
WST	Walk straight and turn
TUG	Timed up and go
ADL	Activities of daily living
GCE	Gait cycle event
IRB	Institutional review board
ROIs	Region of interests
ASI	Absolute symmetry index
TCNN	Temporal convolutional neural network

## References

[1] Zhou Z., Yi L., Wang Q., Lim T.M., Tan E.K. (2023). Role of Dopamine in the Pathophysiology of Parkinson’s Disease. Transl. Neurodegener..

[2] Galván A., Devergnas A., Wichmann T. (2015). Alterations in Neuronal Activity in Basal Ganglia-Thalamocortical Circuits in the Parkinsonian State. Front. Neuroanat..

[3] Jankovic J. (2008). Parkinson’s Disease: Clinical Features and Diagnosis. J. Neurol. Neurosurg. Psychiatry.

[4] Poewe W., Seppi K., Tanner C.M., Halliday G.M., Brundin P., Volkmann J., Schrag A., Lang A.E. (2017). Parkinson Disease. Nat. Rev. Dis. Primers.

[5] Park A., Stacy M. (2011). Dopamine-Induced Nonmotor Symptoms of Parkinson’s Disease. Park. Dis..

[6] Parra E.C., Jiménez M.P.C., Useros M.V.D., Hernández-Martínez A., Molina-Alarcón M. (2021). Relationship Between Motor and Nonmotor Symptoms and Quality of Life in Patients with Parkinson’s Disease. Nurs. Rep..

[7] Alonso-Canovas A., Voeten J., Gifford L., Thomas O., Lees A.J., Bloem B.R. (2023). The Early Treatment Phase in Parkinson’s Disease: Not a Honeymoon for All, Not a Honeymoon at All?. J. Park. Dis..

[8] Isaacson S., Hauser R.A. (2009). Review: Improving Symptom Control in Early Parkinson’s Disease. Ther. Adv. Neurol. Disord..

[9] Munhoz R.P., Tumas V., Pedroso J.L., Silveira-Moriyama L. (2024). The Clinical Diagnosis of Parkinson’s Disease. Arq. Neuro-Psiquiatr..

[10] Zhang Y., Tartaglia M.C., Zhan W., Ofori E. (2023). Editorial: Neuroimaging in Parkinson’s Disease and Parkinsonism. Front. Neurol..

[11] Grover S., Somaiya M., Kumar S., Avasthi A. (2014). Psychiatric Aspects of Parkinson’s Disease. J. Neurosci. Rural Pract..

[12] Rao S.S., Hofmann L.A., Shakil A. (2006). Parkinson’s Disease: Diagnosis and Treatment. Am. Fam. Physician.

[13] Holmes S., Tinaz S. (2024). Neuroimaging Biomarkers in Parkinson’s Disease. Neurophysiologic Biomarkers in Neuropsychiatric Disorders.

[14] Zaman M.S., Ghahari S., McColl M.A. (2021). Barriers to Accessing Healthcare Services for People with Parkinson’s Disease: A Scoping Review. J. Park. Dis..

[15] Meissner W.G. (2012). When Does Parkinson’s Disease Begin? From Prodromal Disease to Motor Signs. Rev. Neurol..

[16] Tolosa E., Garrido A., Scholz S.W., Poewe W. (2021). Challenges in the Diagnosis of Parkinson’s Disease. Lancet Neurol..

[17] Bayen S., Lagon X., Cauet C., Bayen M., Richebe T., Messaadi N., Calafiore M. (2025). Time is Health: Management of Parkinson’s Disease in Primary Care: A Retrospective Quantitative Study of Diagnostic and Therapeutic Timelines. BMC Prim. Care.

[18] Guo Y., Yang J., Liu Y., Chen X., Yang G. (2022). Detection and Assessment of Parkinson’s Disease Based on Gait Analysis: A Survey. Front. Aging Neurosci..

[19] Zhang X., Fan W., Hu Y., Li L., Chen Z., Guan Q. (2022). Single- and Dual-Task Gait Performance and Their Diagnostic Value in Early-Stage Parkinson’s Disease. Front. Neurol..

[20] Yin W., Zhu W., Gao H., Niu X., Shen C., Fan X., Wang C. (2024). Gait Analysis in the Early Stage of Parkinson’s Disease with a Machine Learning Approach. Front. Neurol..

[21] Mirelman A., Bonato P., Camicioli R., Ellis T.D., Giladi N., Hamilton J.L., Hass C.J., Hausdorff J.M., Pelosin E., Almeida Q.J. (2019). Gait Impairments in Parkinson’s Disease. Lancet Neurol..

[22] Fling B.W., Curtze C., Horak F.B. (2018). Gait Asymmetry in People with Parkinson’s Disease Is Linked to Reduced Integrity of Callosal Sensorimotor Regions. Front. Neurol..

[23] Toly Chen T.-C., Lee Y.-J. (2024). Smart and Healthy Walking: Toward Better Health and Life in Smart Cities.

[24] Nassajpour M., Seifallahi M., Rosenfeld A., Tolea M.I., Galvin J.E., Ghoraani B. (2025). Comparison of Wearable and Depth-Sensing Technologies with Electronic Walkway for Comprehensive Gait Analysis. Sensors.

[25] Klöpfer-Krämer I., Brand A., Wackerle H., Müßig J.A., Kröger I., Augat P. (2019). Gait Analysis—Available Platforms for Outcome Assessment. Injury.

[26] Hulleck A.A., Mohan D.M., Abdallah N., El-Rich M., Khalaf K. (2022). Present and Future of Gait Assessment in Clinical Practice: Towards the Application of Novel Trends and Technologies. Front. Med. Technol..

[27] Schlachetzki J.C.M., Barth J., Marxreiter F., Goßler J., Kohl Z., Reinfelder S., Gaßner H., Aminian K., Eskofier B.M., Winkler J. (2017). Wearable Sensors Objectively Measure Gait Parameters in Parkinson’s Disease. PLoS ONE.

[28] Prasanth H., Caban M., Keller U., Courtine G., Ijspeert A.J., Vallery H., von Zitzewitz J. (2021). Wearable Sensor-Based Real-Time Gait Detection: A Systematic Review. Sensors.

[29] Chatzaki C., Skaramagkas V., Tachos N.S., Christodoulakis G., Maniadi E., Kefalopoulou Z., Fotiadis D.I., Tsiknakis M. (2021). The Smart-Insole Dataset: Gait Analysis Using Wearable Sensors with a Focus on Elderly and Parkinson’s Patients. Sensors.

[30] Boucharas D., Androutsos C., Gkois G., Tsakanikas V., Pezoulas V., Manousos D., Skaramagkas V., Chatzaki C., Kontogiannis S., Spandonidis C. (2022). Smart Insole: A Gait Analysis Monitoring Platform Targeting Parkinson Disease Patients Based on Insoles. arXiv.

[31] Biswas N., Chakrabarti S., Jones L., Ashili S. (2023). Smart Wearables Addressing Gait Disorders: A Review. Mater. Today Commun..

[32] Santos V., Gomes B.B., Neto M.A., Amaro A.M. (2024). A Systematic Review of Insole Sensor Technology: Recent Studies and Future Directions. Appl. Sci..

[33] Subramaniam S., Majumder S., Faisal A.I., Deen M.J. (2022). Insole-Based Systems for Health Monitoring: Current Solutions and Research Challenges. Sensors.

[34] Ramamoorthy E.N., Kleiber B. (2015). The Foot.

[35] Perry J., Burnfield J.M. (2024). Gait Analysis.

[36] Herzog W., Nigg B.M., Read L., Olsson E. (1989). Asymmetries in Ground Reaction Force Patterns in Normal Human Gait. Med. Sci. Sports Exerc..

[37] Butterworth P., Urquhart D.M., Landorf K.B., Wluka A.E., Cicuttini F., Menz H.B. (2014). Foot Posture, Range of Motion and Plantar Pressure Characteristics in Obese and Non-Obese Individuals. Gait Posture.

[38] Karamanidis K., Arampatzis A., Bruggemann G.-P. (2003). Symmetry and Reproducibility of Kinematic Parameters during Various Running Techniques. Med. Sci. Sports Exerc..

[39] Fukuchi C.A., Fukuchi R., Duarte M. (2019). Effects of Walking Speed on Gait Biomechanics in Healthy Participants: A Systematic Review and Meta-Analysis. Syst. Rev..

[40] Chen M., Sun Z., Xin T., Chen Y., Su F. (2023). An Interpretable Deep Learning Optimized Wearable Daily Detection System for Parkinson’s Disease. IEEE Trans. Neural Syst. Rehabil. Eng..

[41] Duncanson K.A., Horst F., Abbasnejad E., Hanly G., Robertson W.S.P., Thewlis D. (2024). Modelling Individual Variation in Human Walking Gait across Populations and Walking Conditions via Gait Recognition. J. R. Soc. Interface.

[42] Gupta S., Saviour C.M., Pal B., Chanda S., Mukherjee K. (2025). Biomechanics of Gait. Biomechanics of Gait.

[43] Lee S.-S., Choi S.T., Choi S. (2019). Classification of Gait Type Based on Deep Learning Using Various Sensors with Smart Insole. Sensors.

[44] He K., Zhang X., Ren S., Sun J. Delving Deep into Rectifiers: Surpassing Human-Level Performance on ImageNet Classification. Proceedings of the IEEE International Conference on Computer Vision.

[45] Kimmeskamp S., Hennig E.M. (2001). Heel to Toe Motion Characteristics in Parkinson Patients during Free Walking. Clin. Biomech..

[46] Jones A.D., Crossland S., Nixon J., Siddle H.J., Culmer P., Russell D. (2024). A Cross-Sectional Pilot Study Utilising STRain Analysis and Mapping of the Plantar Surface (STAMPS) to Measure Plantar Load Characteristics within a Healthy Population. Gait Posture.

[47] Xu C., Wen X., Huang L., Shang L., Cheng X., Yan Y., Lei W. (2017). Normal Foot Loading Parameters and Repeatability of the Footscan® Platform System. J. Foot Ankle Res..

[48] Wolff C., Steinheimer P., Warmerdam E., Dahmen T., Slusallek P., Schlinkmann C., Chen F., Orth M., Pohlemann T., Ganse B. (2023). Effects of Age, Body Height, Body Weight, Body Mass Index and Handgrip Strength on the Trajectory of the Plantar Pressure Stance-Phase Curve of the Gait Cycle. Front. Bioeng. Biotechnol..

[49] Rößler R., Wagner J., Knaier R., Rommers N., Kressig R.W., Schmidt-Trucksäss A., Hinrichs T. (2024). Spatiotemporal Gait Characteristics Across the Adult Lifespan: Reference Values from a Healthy Population—Analysis of the COMPLETE Cohort Study. Gait Posture.

[50] Anderson A.J., Eguren D., Gonzalez M., Khan N., Watkinson S.A., Caiola M., Hirczy S., Zabetian C.P., Mills K.A., Moukheiber E. (2024). WearGait-PD: An Open-Access Wearables Dataset for Gait in Parkinson’s Disease and Age-Matched Controls. medRxiv.

[51] Santos V., Gomes B.B., Neto M.A., Rodrigues P.F., Amaro A.M. (2025). A Systematic Review on Smart Insole Prototypes: Development and Optimization Pathways. Actuators.

[52] Fazio R.D., Perrone E., Velázquez R., Di Vittorio M., Visconti P. (2021). Development of a Self-Powered Piezo-Resistive Smart Insole Equipped with Low-Power BLE Connectivity for Remote Gait Monitoring. Sensors.

[53] Neseem M., Nelson J., Reda S. AdaSense: Adaptive Low-Power Sensing and Activity Recognition for Wearable Devices. Proceedings of the 2020 57th ACM/IEEE Design Automation Conference (DAC).

[54] Park J.M., Moon C.-W., Lee B.C., Oh E., Lee J., Jang W., Cho K.H., Lee S.-H. (2024). Detection of Freezing of Gait in Parkinson’s Disease from Foot-Pressure Sensing Insoles Using a Temporal Convolutional Neural Network. Front. Aging Neurosci..

[55] Mun F., Choi A. (2022). Deep Learning Approach to Estimate Foot Pressure Distribution in Walking with Application for a Cost-Effective Insole System. J. NeuroEng. Rehabil..

[56] Leyh C., Feipel V. (2022). Impact of Sex and Velocity on Plantar Pressure Distribution during Gait: A Cross-Sectional Study Using an Instrumented Pressure-Sensitive Walkway. J. Funct. Morphol. Kinesiol..

[57] Varkanitsa M., Peñaloza C., Charidimou A., Kiran S. (2023). Cerebral Small Vessel Disease Burden: An Independent Biomarker for Anomia Treatment Responsiveness in Chronic Stroke Patients with Aphasia. Arch. Phys. Med. Rehabil..

[58] Lee M., Youm C., Noh B., Park H., Cheon S. (2020). Gait Characteristics under Imposed Challenge Speed Conditions in Patients with Parkinson’s Disease during Overground Walking. Sensors.

[59] Raza A., Mahmood I., Sultana T., Sultana S. (2025). Differences in Gait Dynamic Stability of Healthy Subjects Due to Age, Body Mass, Height, BMI, and Walking Speed. Med. Nov. Technol. Devices.

